# Mass spectrometric identification and quantification of the antibiotic clavulanic acid in broiler chicken plasma and meat as a necessary analytical tool in finding ways to increase the effectiveness of currently used antibiotics in the treatment of broiler chickens

**DOI:** 10.1007/s00216-021-03307-6

**Published:** 2021-04-14

**Authors:** Kristina Putecova, Katerina Nedbalcova, Iva Bartejsova, Monika Zouharova, Katarina Matiaskova, Kamil Stastny

**Affiliations:** Veterinary Research Institute, Hudcova 296/70, 62100 Brno, Czech Republic

**Keywords:** Clavulanic acid, Antibiotics, Pharmacokinetics, Tandem mass spectrometry

## Abstract

Clavulanic acid is a molecule with antimicrobial effect used in several livestock species treatment. Its inclusion in the treatment of infectious diseases of broilers requires determination of pharmacokinetic and pharmacodynamic parameters in order to determine the appropriate dosage for broilers and ensure safety of chicken products for human health. The present study describes the optimisation of analytical LC-MS/MS method for identification and quantification of clavulanic acid in broiler chicken plasma and meat. The limit of detection and the limit of quantification for the developed method were 3.09 μg·L^−1^ and 10.21 μg·L^−1^ for plasma and 2.57 μg·kg^−1^ and 8.47 μg·kg^−1^ for meat. The recoveries of the developed plasma and tissue extraction procedure were > 105.7% and > 95.6%, respectively. The achieved coefficient of variation of within-run precision ranged from 2.8 to 10.9% for plasma and from 6.5 to 8.5% for meat. The pharmacokinetic experiment was performed in 112 Ross broiler chickens assigned into time interval groups ranging from 10 min to 24 h in accredited animal facilities. Administered dose of clavulanic acid was 2.5 mg·kg^−1^ according to the manufacturer’s recommendations. The pharmacokinetic parameters obtained from the experiment are as follows: C_max_ = 1.82 ± 0.91 mg·L^−1^, T_max_ = 0.25 h, T_1/2_ = 0.87 h, K_el_ = 0.80 ± 0.04 h^−1^, AUC_0-∞_ = 2.17 mg·h ·L^−1^.

**Supplementary Information:**

The online version contains supplementary material available at 10.1007/s00216-021-03307-6.

## Introduction

Massive, ever-increasing chicken production in the world will not do without the use of antimicrobial agents to prevent massive deaths of chickens due to infectious diseases that can cause large economic and financial damage. However, the emerging occurrence of resistant bacterial strains to these agents represents a risk. Therefore, it is important, in the interest of not only veterinary but also human medicine, to approach the administration of antimicrobial agents to livestock carefully and eliminate the resistance by appropriate selection of antibiotic and duration of administration [1].

Clavulanic acid is an antibiotic with a weak antimicrobial effect. However, its additional ability to inhibit β-lactamase makes it an appropriate supplement to other β-lactam antibiotics, for example amoxicillin and ticarcillin. The mechanism of action of β-lactam antibiotics is based on the inhibition of proteins, especially penicillin binding proteins (PBPs), involved in cell wall formation. PBPs utilise spherically similar β-lactam rings instead of original structures and this causes their blockage and cell wall synthesis interruption. The consequence is an increase of cell wall permeability and subsequent collapse. However, there are bacterial strains that have developed the ability of the enzyme β-lactam ring cleavage as a mechanism of bacterial resistance. Therefore, inhibitors capable of inhibiting this enzyme in order to increase the effect of the administered β-lactam antibiotics are used in antibiotic treatment. The most commonly used β-lactamase inhibitors are tazobactam, sulbactam, and clavulanic acid. The mechanism of β-lactamase inhibition is based on transient acylation of the enzyme’s serine active site followed by hydrolysis of the inhibitor before complete β-lactamase inactivation [2].

The findings mentioned above and beneficial properties of clavulanic acid in bacterial infection treatment increase interest in its usage in veterinary medicine. Antibiotic treatment of farm animals and the presence of residues in products of animal origin are matters of concern for public health. Therefore, the European Community has established Council Directive 96/23/EC [3] that classifies clavulanic acid in group B. To use group B substances in animal treatment, controlling the compliance with MRLs (maximum residue levels) is required. For new drug introduction, it is also necessary to determine pharmacokinetic and pharmacodynamic parameters for the animal species and age that are characteristic of the treated animal. This monitoring requires sensitive and reliable analytical methods for clavulanic acid determination in various matrices. According to Commission Decision 2002/657/EC of 12 August 2002 [4], confirmatory analytical methods are suitable for monitoring antibiotic residues when information on the chemical structure of the analyte is provided, sufficient specificity is enabled, and a suitable internal standard is added. Liquid chromatography in connection with high-resolution mass spectrometry thus seems to be an optimal analytical method for the monitoring of clavulanic acid residues in animal products. A general description of the criteria for the limit of detection (LOD), limit of quantification (LOQ), linearity, and other performance characteristics that have been found to be suitable for the validation of analytical methods used in veterinary drug residue depletion studies is stated in the guidance document VICH GL49 issued by the Committee for Medicinal Products for Veterinary Use [5].

After investigation of the available literature, we found that methods for HPLC separation followed by mass spectrometric detection of clavulanic acid have been mainly developed for human plasma. For example, Yoon et al. [6] developed a clavulanic acid determination method performed on single quadrupole with chromatographic separation on a C8 column. Sample preparation included acetonitrile precipitation followed by dichloromethane extraction. LOD, LOQ, and recovery were 20 μg·L^−1^, 62 μg·L^−1^, and 95.6%, respectively. Zhang et al. [7] conducted a pharmacokinetics study and for its purposes developed a method of clavulanic acid determination utilising triple quadrupole with chromatographic separation on a C18 column. Plasma samples were cleaned up by precipitation in acetonitrile. LLOQ and the recovery were 50 μg·L^−1^ and 97.9–102.4%. Gaikwad et al. [8] developed a clavulanic acid determination method performed on triple quadrupole with chromatographic separation on a C18 column. Human plasma samples were loaded on Oasis HLB SPE columns. LLOQ was 25.28 μg·L^−1^ and the recovery was in the range of 47–51%. For animal plasma, Reyns et al. [9] developed a method for clavulanic acid determination in calf plasma and Yang et al. in cat plasma [10]. Sample preparation included acetonitrile precipitation followed by dichloromethane extraction and syringe filtration, respectively. The detection was performed on a triple quadrupole mass spectrometer with the following validation parameters: LOD, LOQ, and recovery were 3.5 μg·L^−1^, 25 μg·L^−1^, and > 90% for calf plasma and 9 μg·L^−1^, 30 μg·L^−1^, and > 101% for cat plasma.

For tissues, a method for porcine muscle, skin, liver, and kidney with chromatographic separation on a PLRP-S polymeric column and detection on a triple quadrupole mass spectrometer has been developed [11]. The tissue sample was homogenised and purified by ultrafiltration. LOD, LOQ, and recovery were 8–15 μg·kg^−1^, 50 μg·kg^−1^, and > 80%. There are also multiresidual methods including clavulanic acid determination in other human and animal matrices [12, 13].

To our knowledge, no analytical method has been published using LC-HRMS to determine clavulanic acid in chicken plasma or tissue. Jerzsele et al. [14] have determined pharmacokinetic profile after the administration of amoxicillin-clavulanic acid combination at the same dose of clavulanic acid as us (2.5 mg·kg^−1^) using HPLC separation with UV detection. The method was validated on the basis of the calibration curve in HPLC mobile phase and the validation parameters were the following: LLOQ was 80 μg·L^−1^ and recovery >95%.

For reliable quantitative analysis, it is important to find the appropriate internal standard; in the studies mentioned above, terbutaline [6], clenbuterol [7], ampicillin [8], and tazobactam were used [9, 11].

The development and optimisation of an analytical method for the determination of clavulanic acid in various chicken matrices (meat, liver, kidney, plasma) are the first step to be followed in the future by an experiment to determine pharmacokinetic parameters, withdrawal periods, and maximum residue limits according to Regulation (EC) No. 470/2009 of the European Parliament [15] to introduce clavulanic acid into treatment procedures in chicken fattening.

## Materials and methods

### Reagents and materials

The analytical standards of potassium clavulanate (Vetranal™, analytical standard), lithium clavulanate (European Pharmacopoeia Reference Standard), tazobactam sodium salt (analytical standard), and sulbactam sodium salt (European Pharmacopoeia Reference Standard) were purchased from Sigma-Aldrich. Clavulanic acid methyl ester ^13^C D_3_ was purchased from Clearsynth Labs. To prepare standard stock solutions, the analytical standards were dissolved in deionised water. For chromatographic columns and internal standards testing and method conditions optimisation purposes, working solutions were prepared by dilution of stock solution in a mixture of acetonitrile and water. The proportion of acetonitrile varied according to the concentration of acetonitrile used in the initial composition of the mobile phase. While the recommended gradient for Hypercarb and Luna Omega Polar column initiates in low acetonitrile concentration proceeding to higher acetonitrile content, the HILIC column gradient works in the opposite way. Acetonitrile hypergrade for LC-MS LiChrosolv® was purchased from Merck and formic acid was purchased from Sigma-Aldrich. The Pierce ESI negative and positive ion calibration solutions were obtained from Thermo Fisher Scientific (Waltham, MA, USA). Deionised water was prepared with a water treatment system from Goldman water s.r.o. Oasis HLB 3 cc (60 mg) and MAX 3 cc (60 mg) extraction cartridges were purchased from Waters Corporation (Milford, MA). Ultrafilters Vivacon 500 10,000 MWCO were purchased from Sartorius (Göttingen, Germany). The following columns were purchased for chromatographic separation testing: Hypercarb 3 μm 100 × 2.1 mm from Thermo Fisher Scientific (Waltham, MA, USA), Luna Omega 1.6 μm Polar C18 100 × 2.1 mm, and Kinetex 1.7 μm HILIC 100 × 2.1 mm from Phenomenex (Torrance, CA, USA).

### Plasma extraction procedure

To 495 μL of plasma sample, 5 μL of internal standard (tazobactam) was added to final concentration of 100 μg·L^−1^, shortly vortexed and centrifuged to drain the drops from the lid. Protein precipitation was performed by taking 100 μL of the prepared solution added to 100 μL of acetonitrile in a 2-mL centrifugal tube and thorough vortexing for 1 min. After centrifugation (5 min, 4 °C, 20,000 RCF), the supernatant was transferred to a vial and injected into the LC-MS/MS system.

### Tissue extraction procedure

Approximately 20 g of broiler tissue was precisely homogenised with device IKA ULTRA TURRAX T18 basic. To 1 g of homogenised tissue in a 5-mL centrifugal tube, 2 mL of acetonitrile and 10 μL of internal standard (tazobactam) were added to a final concentration of internal standard (100 μg·kg^−1^) and vortexed for 5 min. The mixture was centrifuged (10 min, 4 °C, 14,000 RCF) and placed in the freezer (−20 °C) for 15 min. Subsequently, 400 μL of the supernatant was filtered through centrifugal ultrafilters Vivacon 500 (10,000 MWCO) and filtrate was transferred to a vial and injected into the LC-MS/MS system.

### Chromatographic separation

Chromatographic separation was achieved by gradient elution on the LC system Accela 1200 from Thermo Fisher Scientific (Waltham, MA, USA). The following three different chromatographic columns were tested in our study: Hypercarb 3 μm 100 × 2.1 mm from Thermo Fisher Scientific (Waltham, MA, USA), Luna Omega 1.6 μm Polar C18 100 × 2.1 mm, and Kinetex 1.7 μm HILIC 100 × 2.1 mm from Phenomenex (Torrance, CA, USA). Chromatographic conditions in the test (mobile phases, flow rate, and gradient) were the same for each column except for the composition of the mobile phases, which was for Hypercarb and Luna Omega (the reverse-phase columns) opposite to the HILIC column (normal-phase column). After evaluating the chromatographic separation properties of the tested columns, chromatographic column Kinetex 1.7 μm HILIC 100 × 2.1 mm from Phenomenex (Torrance, CA, USA) was used. The gradient LC system was operated using 0.1% formic acid in water: acetonitrile (95:5, v/v, mobile phase A) and 0.1% formic acid in water: acetonitrile (5:95, v/v mobile phase B) at a flow of 200 μL·min^−1^. A gradient elution for separation on HILIC column was performed: 0–4 min (0% A, 100% B), 8 min (linear gradient to 30% A), 8–12 min (linear gradient to 100% B), and 12–14 min (0% A, 100% B).

### Internal standard selection

The EU regulation 2002/657/EC [4] released in 2002 recommends adding a structurally suitable internal standard at the beginning of the extraction procedure for monitoring sample preparation and measurement. Three internal standards were tested in order to select the most appropriate for our methodology. It is generally known that the most suitable internal standard for mass spectrometric analysis is the isotopically labelled analyte of interest, but it was not available to us. Tazobactam and sulbactam belong to the class of β-lactamase inhibitors such as clavulanic acid. Due to their similar structure, similar chemical properties can be expected, including retention on the column. The third tested internal standard was clavulanic acid methyl ester ^13^C D_3_. The standard solutions were prepared in 90% ACN and analysed on LC-MS/MS.

### Mass spectrometric detection

The analysis was performed on the tandem hybrid mass spectrometer Q Exactive (Thermo Fisher Scientific, USA) equipped with a heated electrospray ionisation probe measured in negative mode (H-ESI-). Before the start of each acquisition series, the mass spectrometer was externally calibrated to the mass accuracy (MA) with negative ion calibration solution. Optimisation of the instrument and collision cell (HCD) parameters was performed by direct syringe infusion of working solutions of 100 μg·L^−1^ of clavulanic acid and tested internal standards with a flow rate of 5 μL min^−1^. The mass spectrometer was operated in the parallel reaction monitoring mode with high-resolution RP = 17,500 (FWHM) at 200 m/z using the following settings: sheath gas flow rate 30 (unit), aux gas flow rate 10 (units), spray voltage 3.00 kV, capillary temperature 210 °C, aux gas heater temperature 200 °C, S-lens RF level 50, AGC target of 6·e^6^, and a maximal inject time of 200 ms. For the identification (confirmation), precursor ions and three product ions for clavulanic acid and the tested internal standards with MA < 5 ppm were measured. The most intense product ion of clavulanic acid and the most compliant internal standard were selected for quantification.

The control of the whole LC-MS system, measured data storage, and processing was performed by using Xcalibur 3.1 software and data evaluation for identification purposes was performed by using Mass Frontier v. 7.0.

### Validation parameters

The presented analytical method was validated by a set of parameters that followed the recommendations as defined by the Commission Decision 2002/657/EC [4] and by the European Medicines Agency in VICH-GL49 [5].

For generating standard calibration curves, control matrices (acetonitrile, plasma, meat) were fortified with clavulanic acid (at 6 different concentration levels in the range 10–2000 μg·L^−1^ in plasma and acetonitrile, at 7 different concentration levels in the range 50–2000 μg·kg^−1^ in meat) and internal standard (100 μg·L^−1^ in plasma and acetonitrile, 100 μg·kg^−1^ in meat) before the extraction procedure. Fortification of plasma samples and acetonitrile solutions was easily performed by adding an appropriate amount of clavulanic acid standard to the matrix to achieve individual concentration levels. In case of meat, clavulanic acid standard solution was added to homogenised tissue and precisely vortexed after acetonitrile addition. Each concentration level was performed in triplicate. Linearity was described according to the document VICH-GL49 [5] using weighted linear regression plot of peak area ratio vs known analyte concentration using weighting factor $\frac{1}{\mathrm{x}}$. Correlation coefficient (R^2^), slope, and intercept with appropriate standard deviations were obtained from regression equation of the calibration curve.

For LOD determination, a method based on analysis of 10 blank samples spiked with the lowest concentration level of the calibration curve (10 μg·L^−1^ for plasma and acetonitrile, 50 μg·kg^−1^ for meat) was used. LOD was calculated using the equation $LOD=3.9\ \frac{\mathrm{SD}\ \mathrm{of}\ \mathrm{spiked}\ \mathrm{sample}\ \mathrm{signals}}{\mathrm{slope}}$ stated in EU guidance document on the estimation of LOD and LOQ [16]. Also, LOQ was calculated as this guideline recommends multiplying LOD by 3.3.

The accuracy of the analytical method was expressed as the average percentage recovery obtained by comparison of measured value of each sample concentration level of calibration curve against expected concentration according to the formula:$\mathrm{Recovery}\ \left(\%\right)=\frac{\mathrm{Measured}\ \mathrm{concentration}}{\mathrm{Expected}\ \mathrm{concentration}}\ast 100$. The accuracy calculation was based on the requirements of the document VICH-GL49 [5] and recovery should not be out of the following acceptable range: −20% to +10% of expected concentration.

Single-laboratory validation precision was performed according to recommendations in VICH-GL49 [5] including within-run precision (repeatability). For the evaluation of precision of the method, three replicates for three different concentrations from the calibration scale (10, 500, 2000 μg·L^−1^ for plasma and acetonitrile, 50, 400, 2000 μg·kg^−1^ for meat) were used. According to the guideline, the obtained coefficient of variation (CV) for within-run precision for the concentration range of clavulanic acid used in our study should not exceed the value of 15% for concentration levels 10 and 50 μg·L^−1^ (μg·kg^−1^ in meat) and 10% for concentration levels ≥100 μg·L^−1^ (μg·kg^−1^ in meat).

Subsequently, the selectivity and specificity of the method were evaluated. As reported by Holčapek et al. [17], Orbitrap is one of the ultrahigh-resolution mass analysers providing excellent selectivity and specificity due to the possibility of obtaining very narrow peaks. When external calibration is used, the MA can reach below 3 ppm. Specificity and selectivity were evaluated by comparing 20 blank samples and 20 fortified samples of each matrix (plasma, meat). The specificity is limited by the existence of structural isomerism and stereoisomerism of the analyte. However, structural isomers can be distinguished by optimal fragmentation energy generating isomer-specific fragments. We use three fragments for clavulanic acid identification to eliminate structural isomer substitution: 136.04040, 108.04549, 82.02984. The stereoisomers can be separated in time by gradient liquid chromatography.

### Stability

There are several publications reporting high instability of clavulanic acid under various conditions that must be taken into account when developing the analytical method and analysing samples. Bersanetti et al. [18] observed that the stability of clavulanic acid rapidly decreases as temperature increases. The stability of clavulanic acid can also be affected by the pH value. Santos et al. [19] confirmed the findings that pH around 6 is optimal for clavulanic acid and more acidic or alkaline conditions increase its instability. In addition, the authors also came to the conclusion that the degradation of clavulanic acid is a function of the ionic strength and it increased in the presence of all tested salts compared to the buffer alone. Instability of clavulanic acid in methanol solution [11] also complicates sample processing because of exclusion of methanol added to storage, extraction, and chromatographic solvents. Therefore, in the developed method, acetonitrile was used instead of methanol.

In our study, stability of clavulanic acid was tested according to recommendations as defined by the European Medicines Agency in VICH-GL49 [5]. In order to determine the appropriate storage conditions and the length of time the samples (plasma, muscle) can be stored, the spiked samples (100, 200, 1000, 2000 μg·L^−1^, μg·kg^−1^, respectively) were analysed in triplicate after storage at 4 °C, −20 °C, and − 80 °C for 1, 2, 4, and 12 weeks. Under certain circumstances, the processed samples can be assayed after a few hours or on the next day. Therefore, the stability of clavulanic acid was also determined in solutions with concentrations 100, 200, 1000, 2000 μg·L^−1^, μg·kg^−1^, respectively, in triplicate after 48 h at 4 °C and after 4 and 8 h at room temperature in the dark and in the light.

### Pharmacokinetic experiment

The developed analytical method was used in practice in a pharmacokinetic experiment for enhancing knowledge about the course of clavulanic acid in the chicken organism. The study was performed in 112 Ross broiler chickens in the accredited animal facilities of the Veterinary Research Institute in Brno according to an animal experiment project approved by the Branch Commission for Animal Welfare of the Ministry of Agriculture of the Czech Republic (permission MZe 2069). Fourteen-day-old chickens were allocated into 14 groups of 8 birds each and the commercially used preparation Amoksiklav (Elanco Europe Ltd., UK) was administered orally according to the manufacturer’s recommendations. The recommended dose of the preparation is 2.5 mg·kg^−1^ and clavulanic acid content in the preparation is 125 g·kg^−1^. The weight of 14-day-old broilers used in our experiment was approximately 0.4 kg. Therefore, 2 mL of solution with concentration of the preparation 5 g·L^−1^ made up of Amoksiklav powder dissolved in drinking water was administered by an oral probe into the throat of the broilers. To obtain pharmacokinetic parameters, chickens after inhalation anaesthesia with isoflurin 1000 g·kg^−1^ (Vetpharma AH, Spain) were sacrificed by decapitation by groups at the following intervals: 0.08, 0.2, 0.25, 0.5, 0.75, 1, 1.5, 2, 3, 4, 5, 6, 12, and 24 h after the drug administration. Two control chickens without Amoksiklav administration were added to the experiment in order to gain blank matrices for extraction procedure development, calibration curve determination, and obtaining validation parameters. Plasma was gained from blood after heparin addition (50 U·mL^−1^) followed by centrifugation (15 min, 4 °C, 1300 RCF) and freezing to −80 °C. Meat from breast muscle was also cut and frozen at −80 °C until processing in order to gain information about stored residues of clavulanic acid in the tissue. Plasma and meat were processed according to plasma and tissue extraction procedure described in previous paragraphs and analysed by LC-MS/MS system. When processing the samples, it was necessary to take into account low stability of clavulanic acid. Therefore, the samples were processed within a week after blood and tissue collection and analysed by LC-MS/MS immediately after sample preparation.

Subsequently, pharmacokinetics of clavulanic acid in broiler chickens was graphically estimated from the pharmacokinetic curve constructed from the obtained data as a mean of the time interval concentration-time dependence. For a better and more detailed understanding of pharmacokinetics of clavulanic acid, pharmacokinetic parameters were derived from the constructed pharmacokinetic curve. Maximum concentration (C_max_) and time the maximum concentration was reached (T_max_) were directly read from pharmacokinetic curve. The elimination constant (K_el_) as a negative value of the slope of the natural logarithm of concentration-time dependence of the elimination part of pharmacokinetic curve and biological half-life (T_1/2_) as 0.693/K_el_ were calculated to describe the elimination rate of clavulanic acid from the chicken body. Total drug exposure over time, expressed as the area under the pharmacokinetic curve (AUC), was estimated by the trapezoidal rule method calculation.

## Results and discussion

### Internal standard selection

Out of all tested internal standards, retention time of clavulanic acid methyl ester ^13^C D_3_ is best suited to retention time of clavulanic acid. However, low stability and response were observed. It has also been shown that clavulanic acid methyl ester ^13^C D_3_ standard solution contains a small portion of clavulanic acid distorting the results. Both tazobactam and sulbactam provide sufficient response; however, retention time of tazobactam is better suited to retention time of clavulanic acid. For the reasons mentioned above, we decided to use tazobactam as internal standard in subsequent measurements.

### Identification (confirmation) of clavulanic acid

Identification was performed in compliance with the recommendations as defined by the Commission Decision 2002/657/EC [4] and confirmation outcomes of clavulanic acid together with the internal standard confirmation outcomes are presented in Table 1. Mass spectrometric detection was performed in negative ESI mode producing precursor ion of clavulanic acid 198.04080 and product ions 136.04040, 108.04549, and 82.02984. Fragmentation energy was set to 17 eV. Mass accuracy, calculated according to the formula $MA=\frac{\frac{m}{z}\exp +\frac{m}{z}\  theor\ }{\frac{m}{z}\  theor}$, was <2 ppm for precursor ion and < 4 ppm for product ions.

**Table 1:** Identification and confirmation data from LC-MS/MS analysis

	Clavulanic acid			Tazobactam (IS)		
Elemental composition	[C_8_H_8_NO_5_]^−^			[C_10_H_11_N_4_O_5_S]^−^		
m/z of precursor ion	198.04080			299.04556		
Mass accuracy of precursor ion (ppm)	−1.62			0.10		
Fragmentation energy (eV)	17			30		
m/z of qualifier product ion	136.04040	82.02984		255.05573	207.08875	
m/z of quantifier product ion			108.04549			138.05605
Mass accuracy of product ion (ppm)	−2.65	−3.41	−1.20	0.74	0.29	0.65
ESI mode	negative			negative		
Retention time (min)	Acetonitrile	Plasma	Meat	Acetonitrile	Plasma	Meat
	1.34	1.26	1.29	1.38	1.27	1.38

### Plasma and tissue extraction procedure

Several plasma extraction procedures were tested in order to achieve optimal results. Firstly, the solid-phase extraction was tested on Oasis HLB and MAX columns according to the manufacturer’s instructions. Retention on HLB columns was found to be ineffective, probably due to polarity of clavulanic acid. Reyns et al. [9] also confirmed this claim after testing HLB columns. Gaikwad et al. [8] developed a clavulanic acid determination method using HLB extraction procedure but with recovery of only about 50%.

On MAX columns, the following four variations of eluates were tested: A (2% HCOOH in methanol), B (2% HCOOH in 45% methanol), C (4% HCOOH in methanol), and D (4% HCOOH in 45% methanol). Only eluent D contains a sufficient quantity of clavulanic acid to be determined, but sensitivity was not satisfactory. The reason of low response was probably low pH of the eluate causing clavulanic acid degradation.

Extraction procedure with acetonitrile precipitation proved to be the most effective. It is in accordance with other publications [6, 7, 9, 10] in which the authors stated recovery more than 90%. Firstly, we tried to concentrate the final sample solution by nitrogen steam evaporation. However, nitrogen steam evaporation to dryness increased clavulanic acid degradation. Therefore, we decided to skip this step.

A rapid and simple tissue extraction procedure without high-temperature application provided satisfactory results despite the high instability of clavulanic acid.

The recoveries of the finally developed plasma and tissue extraction procedure were > 105.7% and < 95.6%, respectively.

We also had a standard solution of the lithium salt of clavulanic acid at our disposal and, compared to potassium salt, it appeared to be more stable, better extractable from plasma and meat, and more suitable to measure by LC-MS/MS. However, in biological tissues, we primarily assumed the presence of potassium salts instead of lithium salts and, therefore, for practical use of the results of this study, it is important to deal with the problems of potassium salt.

### Chromatographic separation

The following three different chromatographic columns were tested in our study: Hypercarb 3 μm 100 × 2.1 mm from Thermo Fisher Scientific (Waltham, MA, USA), Luna Omega 1.6 μm Polar C18 100 × 2.1 mm, and Kinetex 1.7 μm HILIC 100 × 2.1 mm from Phenomenex (Torrance, CA, USA). The column Luna Omega Polar C18 provided a lower response compared to the other tested columns (5 times lower response than Hypercarb column and 7 times lower than HILIC column). The response provided by column Hypercarb was satisfactory but its durability was low in our conditions and, after a few weeks, chromatographic separation was not sufficient. Finally, Kinetex HILIC column with core-shell particles was tested and due to the highest resulting efficiency and sensitivity among the tested columns we used it in further measurements.

### Validation parameters

#### Linearity

The parameters of the calibration curves are given in Table 2 and have the following values: Correlation coefficients calculated for clavulanic acid are ≥0.9929 for acetonitrile, ≥ 0.9991 for plasma, and ≥ 0.9918 for meat. The calculated values of slope and intercept were as follows: slope m = 0.0064 and intercept b = −0.0089 for acetonitrile, m = 0.0113 and b = −0.1093 for plasma, and m = 0.0036 and b = 0.0060 for meat. The estimated values of LOD and LOQ were 3.18 μg·L^−1^ and 10.48 μg·L^−1^ for acetonitrile, 3.09 μg·L^−1^ and 10.21 μg·L^−1^ for plasma, and 2.57 μg·kg^−1^ and 8.47 μg·kg^−1^ for meat.

**Table 2:** Summary of validation parameters obtained from clavulanic acid calibration curves in ACN, plasma, and meat

	ACN			Plasma			Meat		
Linearity									
Concentration range (μg·L^−1^/μg·kg^−1^)	10–2000			10–2000			50–2000		
Number of points	6			6			7		
Correlation coefficient, R^2^	0.9929			0.9991			0.9918		
Standard deviation of the correlation coefficient	0.2857			0.1998			0.2217		
Slope, m	0.0064			0.0113			0.0036		
Standard deviation of the slope, Sm	0.00059			0.00034			0.00008		
Intercept, b	−0.0089			−0.1093			0.0060		
Standard deviation of the intercept, Sb	0.0974			0.0587			0.0688		
LOD (μg·L^−1^/μg·kg^−1^)	3.18			3.09			2.57		
LOQ (μg·L^−1^/μg·kg^−1^)	10.48			10.21			8.47		
Accuracy									
Recovery (%)	94.6			105.7			95.6		
Within-run precision									
Concentration level (μg·L^−1^/μg·kg^−1^)	10	500	2000	10	500	2000	50	400	2000
Calculated concentration (μg·L^−1^/μg·kg^−1^)	7.71	513.73	2076.28	12.3	518.96	2088.70	45.58	399.18	1955.81
CV (%)	13.6	6.5	7.7	10.9	2.8	5.3	8.5	6.5	6.9

In the available literature, most of the developed tandem mass spectrometric methods for clavulanic acid determination are in human plasma with LOD = 20 μg·L^−1^ [6] and LOQ = 25–62 μg·L^−1^ [6–8]. Of the animal plasma methods, there were published methods for calf plasma with LOD = 3.5 μg·L^−1^, LOQ = 25 μg·L^−1^ [9], and cat plasma with LOD = 9 μg·L^−1^, LOQ = 30 μg·L^−1^ [10]. Reyns et al. developed a mass spectrometric method for several types of porcine tissues with LOD = 8–15 μg·kg^−1^ and LOQ = 50 μg·kg^−1^ [11]. However, to our knowledge, a tandem mass spectrometric method for determining clavulanic acid for chicken plasma and tissue has not yet been available. Jerszele et al. determined clavulanic acid in chicken plasma using HPLC separation with UV detection with LLOQ = 80 μg·L^−1^ [14].

#### Accuracy

Accuracy was expressed as the average percentage recovery and resulting values have met the acceptable range listed in guideline VICH-GL49 [5]: −20% to +10% of expected concentration. Recoveries were 94.6% for acetonitrile, 105.7% for plasma, and 95.6% for meat.

#### Precision

According to the recommendations in guideline VICH-GL49 [5], within-run precision is evaluated by CV. Calculated CV for acetonitrile is 13.6% for 10 μg·L^−1^, 6.5% for 500 μg·L^−1^, and 7.7% for 2000 μg·L^−1^. For plasma, calculated values of CV are 10.9% for 10 μg·L^−1^, 2.8% for 500 μg·L^−1^, and 5.3% for 2000 μg·L^−1^. And finally, for meat, calculated values of CV are 8.5% for 50 μg·L^−1^, 6.5% for 400 μg·L^−1^, and 6.9% for 2000 μg·L^−1^. Detailed information is given in overview Table 2. As mentioned in the guideline, CV for the concentration range of clavulanic acid used in our study should not exceed the value of 15% for concentration levels 10 and 50 μg·L^−1^ (μg·kg^−1^ in meat) and 10% for concentration levels ≥100 μg·L^−1^ (μg·kg^−1^ in meat) and our results are clearly below this value.

#### Specificity and selectivity

**Fig. 1:**
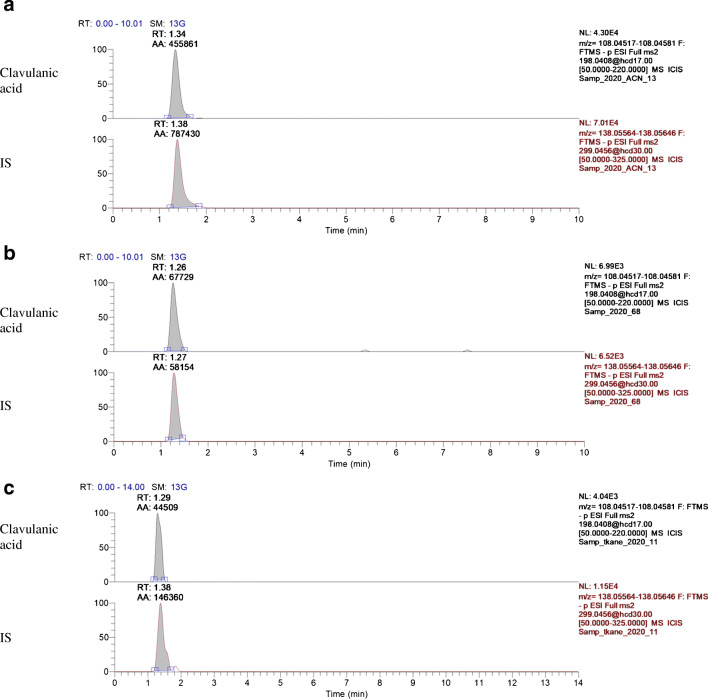
LC-ESI(-)-MS/MS chromatograms of various matrices spiked with standard solution of clavulanic acid (100 μg·L^−1^, 100 μg·kg^−1^ respectively) and internal standard (100 μg·L^−1^, 100 μg·kg^−1^ respectively): **a**, acetonitrile; **b**, broiler chicken plasma; **c**, broiler chicken meat

Mass spectrometer with Orbitrap analyser enables identifying the molecules of interest with high selectivity and specificity as mentioned above. As shown in the chromatogram (Fig. 1 in the article and Fig. S1 in Supplementary Information (ESM)), fortified samples provide narrow peaks of the main product ions in specific retention time without any interference and MA below 3 ppm.

### Stability

**Fig. 2:**
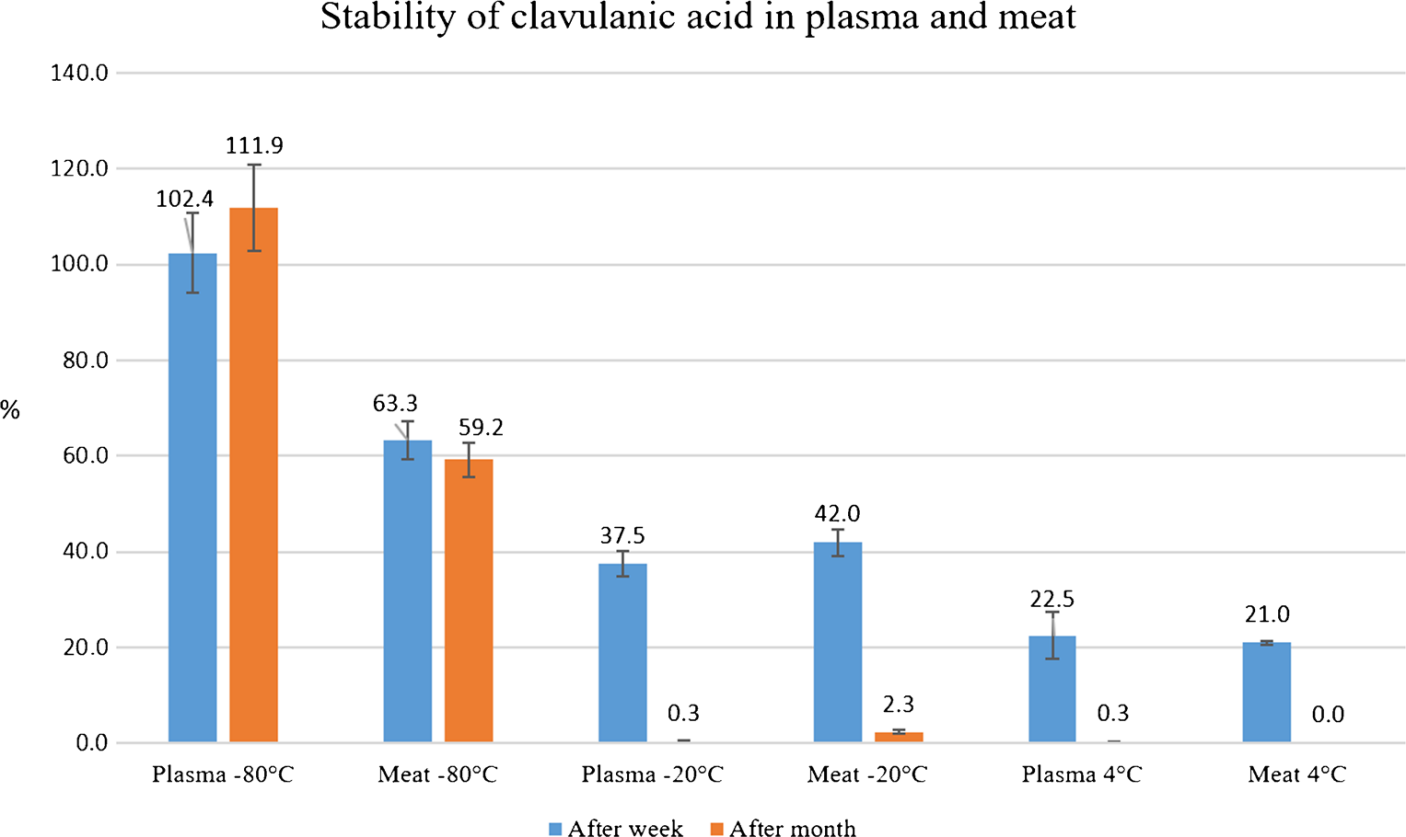
Relative expression of results from stability test of clavulanic acid under various conditions

The stability test confirmed high instability of clavulanic acid in broiler chicken matrices under various conditions. The stability test was performed mainly to confirm high instability of clavulanic acid in our conditions and improve details in sample preparation. The most important outcome is that the concentration of clavulanic acid in plasma and meat at 4 °C and − 20 °C decreases rapidly after only a week. Storage of samples at these temperatures is therefore inappropriate. Stability is higher at −80 °C, but it is necessary to process plasma and meat within a month from sampling if the samples are stored at this temperature. Brief information is given in Fig. 2 and detailed information is given in ESM (Tables S1 and S2). Stability in solution at 4 °C is sufficient after 48 h, as well as at 20 °C after 8 h in the dark and in the light, respectively. Therefore, the sample can be measured 2 days after processing when stored in a refrigerator and within 8 h from processing at room temperature. However, based on the instability obvious from the other measurements, it is obvious that the time from sampling to the measurement is required to be reduced to a minimum for the most accurate results.

Instability of clavulanic acid found in this study corresponds with stability data published by Bersanetti et al. who demonstrated that the stability of clavulanic acid decreases with increasing temperature [18].

### Practical outputs

**Fig. 3:**
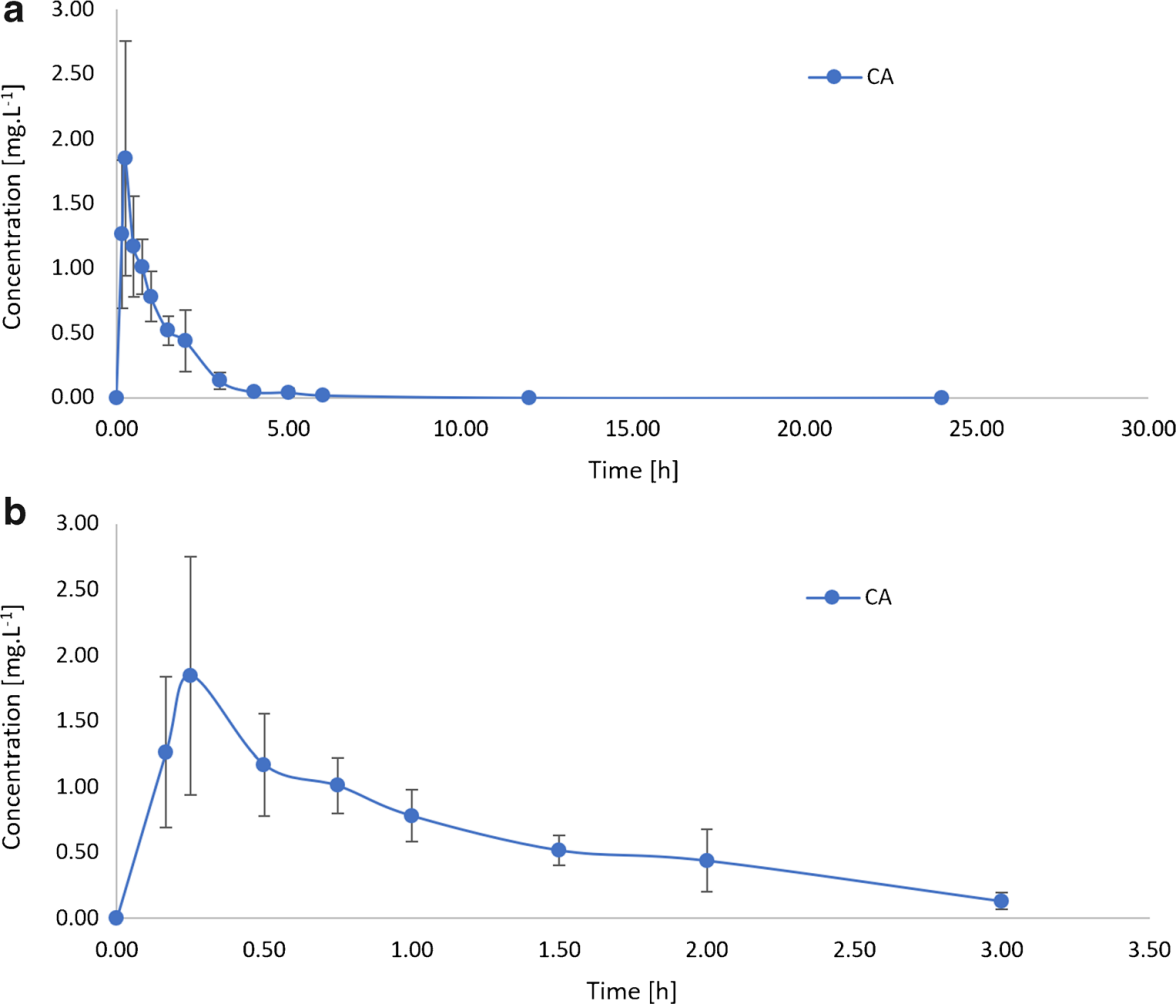
Mean ± SD clavulanic acid plasma concentrations after oral administration to 14-day-old broiler chickens at a dose of 2.5 mg·kg^−1^. Graph **a**, Pharmacokinetic curve in the range of 0–24 h. Graph **b**, Pharmacokinetic curve in the range of 0–3 h

The developed method for clavulanic acid determination in plasma and meat was practically used in a pharmacokinetic experiment performed in 112 broilers. Figure 3 represents the resulting pharmacokinetic curve. Because of improbably high plasma concentration of clavulanic acid, the result from the first time interval (5 min) was excluded from the final pharmacokinetic curve. A very high variability between individual measurements (standard deviation) in the first time interval group was also observed. We assume that the unexpected high concentration values and their variability in the first time interval group were caused by the large volume of the administered dose which was not absorbed from the stomach in such a short time interval and was poured out of the throat during decapitation and blood collection. Therefore, the pharmacokinetic curve and subsequent pharmacokinetic parameters were calculated from data omitting the 5-min time interval (graphs A, B). Reduction of the administered dose volume was limited by low solubility of the preparation.

Calculated pharmacokinetic parameters are summarised in Table 3. The maximum serum concentration C_max_ = 1.82 ± 0.91 mg·L^−1^ was reached at the time interval of 0.25 h after oral administration (T_max_) and dropped to one-half of its value after 0.87 h (T_1/2_). The rate of clavulanic acid elimination from the broiler body is expressed by the estimated elimination constant K_el_ = 0.80 ± 0.04 h^−1^ and the total drug exposure across time is described by the calculated area under the pharmacokinetic curve AUC_0-∞_ = 2.17 mg·h·L^−1^.

**Table 3:** Pharmacokinetic parameters after oral administration to 14-day-old broiler chickens at a dose of 2.5 mg·kg^−1^

Parameter	Value	Unit
C_max_	1.82 ± 0.91	mg·L^−1^
T_max_	0.25	h
T_1/2_	0.87	h
K_el_	0.80 ± 0.04	h^−1^
AUC_0–1440_	2.12	mg·h·L^−1^
AUC_1440-∞_	0.05	mg·h·L^−1^
AUC_0-∞_	2.17	mg·h·L^−1^

Compared to a study by Jerzsele et al. [14] using UV detection technique, C_max_ = 1.08 ± 0.05 mg·L^−1^ and AUC =2.33 ± 0.05 mg·h·L^−1^ have similar values. Small differences were observed for T_max_ = 0.52 ± 0.04 h and T_1/2_ = 1.13 ± 0.06 h parameters, which could have been caused by different ages of the experimental broilers (6 weeks vs. 2 weeks in our study). Administrated dose of clavulanic acid was the same as in our experiment (2.5 mg·kg^−1^). Chickens at an early age are prone to infection more than after a few weeks from the birth. Therefore, it is more appropriate to determine pharmacokinetic curve in the earlier age for the most accurate dosage specification.

The pharmacokinetic parameters of clavulanic acid in other animal species and human described by other authors are comparable. Pharmacokinetic studies were also performed in turkeys, pigs, sheep, and goats with resulting T_max_ = 0.48 h for turkeys, 0.88 h for pigs, and T_1/2_ = 1.27 h for turkeys, 0.67 h for pigs, 1.16 h for sheep, and 0.85 h for goat [20–22]. In humans, values of T_max_ and T_1/2_ are approximately 1 h as mentioned by Todd and Benfield [23].

Clavulanic acid was also measured in chicken meat but was not detected. We assume that the polarity of the clavulanic acid molecule was because it was rapidly excreted in the urine without the possibility of storage in tissues, which is a positive aspect for chicken meat consumers.

Infections in chicken farms can cause massive death and economic damage to breeders, so in most cases it is necessary to prevent these infections by administering antibiotics. However, due to the growing resistance to antibiotics in human and veterinary medicine, it is important to prefer usage of first choice antibiotics and to understand the pharmacokinetics and pharmacodynamics of the drug for proper dosing so that meat intended for human consumption does not contain residues. The results of our pharmacokinetic study can help in the implementation of the drug containing clavulanic acid in the chicken breeding practice.

## Supplementary information

ESM 1 (DOCX 163 kb).

## Data Availability

The data that support the findings of this study are available on request from the corresponding author.
