# Supplementary material for: Mass spectrometric identification and quantification of the antibiotic clavulanic acid in broiler chicken plasma and meat as a necessary analytical tool in finding ways to increase the effectiveness of currently used antibiotics in the treatment of broiler chickens
Source: Anal Bioanal Chem. 2021 Apr 14;413(13):3561–71. doi: 10.1007/s00216-021-03307-6 (PMC8105215; doi:10.1007/s00216-021-03307-6)
Supplement: Supplementary file 1 — (DOCX 163 kb). [file 216_2021_3307_MOESM1_ESM.docx]

**Analytical and Bioanalytical Chemistry**

**Supplementary Information**

**Mass spectrometric identification and quantification of the antibiotic clavulanic acid in broiler chicken plasma and meat as a necessary analytical tool in finding ways to increase the effectiveness of currently used antibiotics in the treatment of broiler chickens.**

Kristina Putecova^1,*^, Katerina Nedbalcova^1^, Iva Bartejsova^1^, Monika Zouharova^1^, Katarina Matiaskova^1^, Kamil Stastny^1^

^1^Veterinary Research Institute, Hudcova 296/70, 62100 Brno, Czech Republic

*****Correspondence: [putecova@vri.cz](mailto:putecova@vri.cz)

Table S1: Results from stability test of clavulanic acid in acetonitrile under various conditions

| **Stability of clavulanic acid in acetonitrile** | | | | | | |
| --- | --- | --- | --- | --- | --- | --- |
| Concentration level [µg.L^-1^] | after 4 hours | | after 8 hours | | after 48 hours | |
|  | % | SD % | % | SD % | % | SD % |
| **solution at 4°C** | | |  |  |  |  |
| 100 | - | - | - | - | 100.0 | 6.5 |
| 200 |  |  |  |  | 90.0 | 10.1 |
| 1000 |  |  |  |  | 82.8 | 6.8 |
| 2000 |  |  |  |  | 93.5 | 9.4 |
| **solution at 20°C in the dark** | | |  |  |  |  |
| 100 | 100.1 | 12.7 | 94.9 | 6.8 | - | - |
| 200 | 104.8 | 3.2 | 109.0 | 4.4 |  |  |
| 1000 | 100.6 | 13.7 | 107.9 | 4.5 |  |  |
| 2000 | 81.0 | 7.2 | 89.4 | 4.8 |  |  |
| **solution at 20°C in the light** | | |  |  |  |  |
| 100 | 94.1 | 5.8 | 84.1 | 5.5 | - | - |
| 200 | 96.7 | 5.8 | 95.3 | 9.8 |  |  |
| 1000 | 82.7 | 7.4 | 96.2 | 5.7 |  |  |
| 2000 | 93.5 | 1.4 | 108.8 | 5.1 |  |  |

Table S2: Results from stability test of clavulanic acid in plasma and meat under various conditions

| **Stability of clavulanic acid in matrices** | | | | | | | | |
| --- | --- | --- | --- | --- | --- | --- | --- | --- |
| Concentration level [µg.L^-1^] | after 1 week | | after 2 weeks | | after 4 weeks | | after 3 months | |
|  | % | SD % | % | SD % | % | SD % | % | SD % |
| **plasma at 4°C** | | |  | | | | | |
| 100 | 22.3 | 9.1 | < LOQ | | < LOQ | | < LOQ | |
| 200 | 22.4 | 4.4 | 9.2 | 4.3 | < LOQ | | < LOQ | |
| 1000 | 25.6 | 2.8 | 11.5 | 1.9 | < LOQ | | < LOQ | |
| 2000 | 19.5 | 3.4 | 7.0 | 2.2 | 0.5 | 0.3 | < LOQ | |
| **plasma at -20°C** | | |  | | | | | |
| 100 | 39.9 | 1.8 | 18.3 | 3.4 | < LOQ | | < LOQ | |
| 200 | 35.1 | 1.8 | 15.8 | 1.1 | < LOQ | | < LOQ | |
| 1000 | 40.4 | 4.3 | 19.1 | 0.4 | < LOQ | | < LOQ | |
| 2000 | 34.7 | 2.8 | 15.6 | 2.3 | < LOQ | | < LOQ | |
| **plasma at -80°C** | | |  | | | | | |
| 100 | 106.7 | 7.7 | 114.2 | 10.5 | 115.8 | 5.5 | 60.1 | 3.8 |
| 200 | 102.1 | 6.6 | 113.1 | 9.5 | 114.4 | 8.3 | 65.5 | 10.0 |
| 1000 | 103.5 | 11.7 | 115.6 | 12.0 | 112.3 | 11.7 | 64.2 | 6.6 |
| 2000 | 97.2 | 7.3 | 113.4 | 2.4 | 104.9 | 10.6 | 61.7 | 12.2 |
| Concentration level [µg.Kg^-1^] | after 1 week | | after 2 weeks | | after 4 weeks | | after 3 months | |
|  | % | SD % | % | SD % | % | SD % | % | SD % |
| **meat at 4°C** | | |  | | | | | |
| 100 | 42.8 | 0.1 | < LOQ | | < LOQ | | < LOQ | |
| 200 | 27.0 | 0.9 | < LOQ | | < LOQ | | < LOQ | |
| 1000 | 10.5 | 0.6 | 4.9 | 0.1 | < LOQ | | < LOQ | |
| 2000 | 3.6 | 0.4 | < LOQ | | < LOQ | | < LOQ | |
| **meat at -20°C** | | |  | | | | | |
| 100 | 46.0 | 2.0 | < LOQ | | < LOQ | | < LOQ | |
| 200 | 36.9 | 4.2 | 13.3 | 9.4 | < LOQ | | < LOQ | |
| 1000 | 51.0 | 3.2 | 21.5 | 4.1 | 7.9 | 0.6 | < LOQ | |
| 2000 | 34.0 | 1.9 | 6.8 | 1.0 | 1.2 | 0.9 | < LOQ | |
| **meat at -80°C** | | |  | | | | | |
| 100 | 70.9 | 4.6 | 74.2 | 2.8 | 66.1 | 2.6 | 42.9 | 1.3 |
| 200 | 52.4 | 1.8 | 56.0 | 3.3 | 51.6 | 3.5 | 29.6 | 1.8 |
| 1000 | 67.8 | 2.4 | 72.7 | 2.6 | 69.5 | 7.8 | 27.0 | 2.4 |
| 2000 | 62.2 | 6.6 | 54.7 | 4.4 | 49.5 | 0.0 | 17.9 | 1.1 |


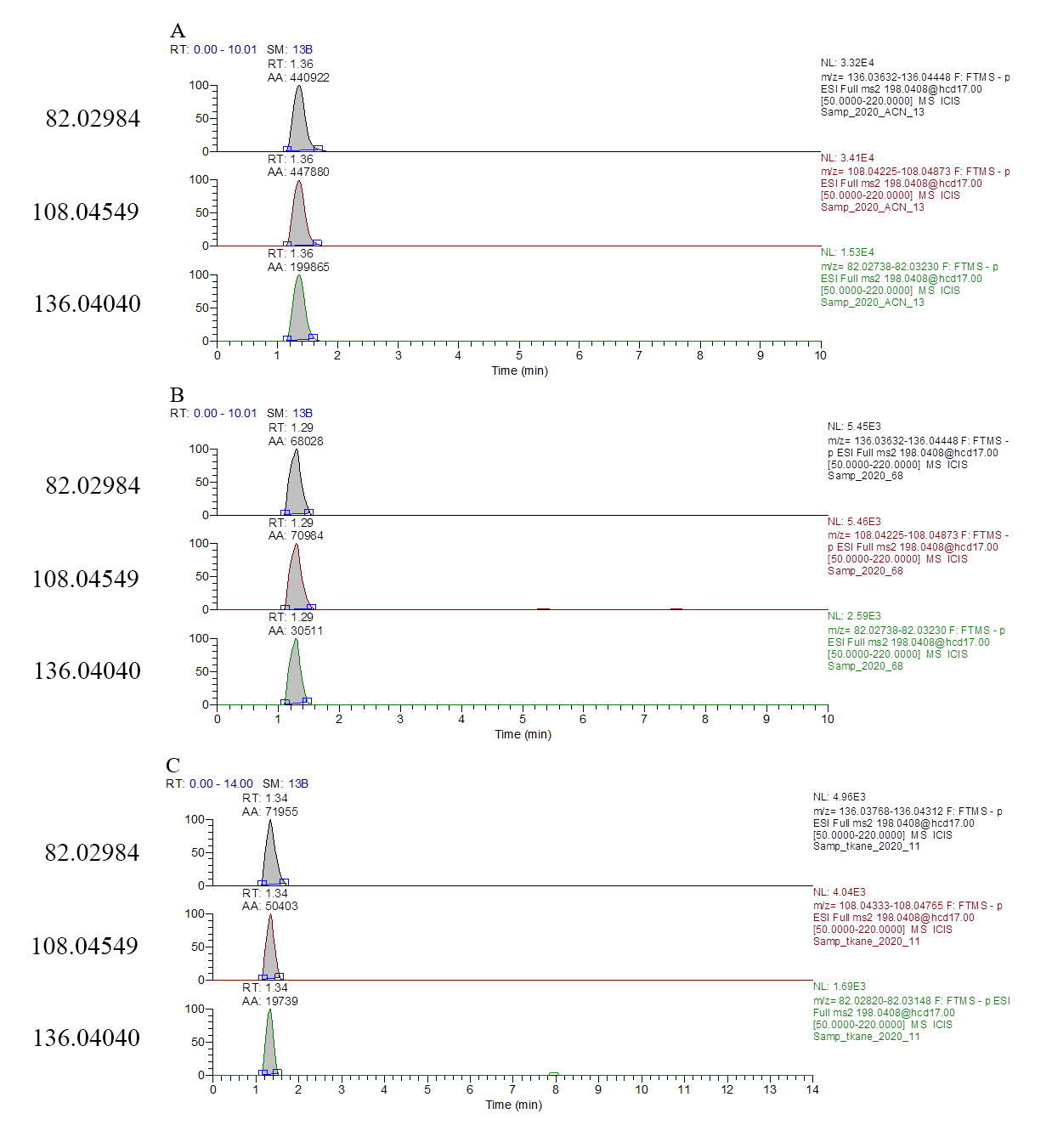


Figure S1: LC-ESI(-)-MS/MS chromatograms of monitored transitions 82.02984, 108.04549 (quantifier), 136.04040 in various matrices spiked with standard solution of clavulanic acid (100 µg.L^-1^) : A-acetonitrile, B- broiler chicken plasma, C- broiler chicken meat
